# Bilateral Orbital Reconstruction With Autologous Bone Graft After Gunshot Wound to Upper Midface

**DOI:** 10.7759/cureus.13611

**Published:** 2021-02-28

**Authors:** Adam Bicak, Jessica Hale, Kristopher M Day

**Affiliations:** 1 Surgery, Marshall University Joan C. Edwards School of Medicine, Huntington, USA; 2 General Surgery, Marshall University Joan C. Edwards School of Medicine, Huntington, USA; 3 Plastic Surgery, University of Iowa Hospitals and Clinics, Iowa City, USA

**Keywords:** orbit fracture, trauma, plastic surgery

## Abstract

Ballistic maxillofacial injuries are highly destructive, producing significant morbidity and mortality. Survivors’ defects pose unique reconstructive challenges, such as loss of periorbital bone stock in upper midface injuries. While orbital reconstruction has transitioned from primarily autologous grafts to alloplastic implants, ballistic trauma remains a niche that warrants the use of autologous bone equally with alloplastic materials. We report a case of an upper midface gunshot wound in a 20-year-old male producing bilateral comminuted medial orbital wall fractures. Reconstruction utilized bilateral split-thickness calvarial bone grafts through preseptal transcaruncular transconjunctival incisions. This case illustrates the utility of autologous bone grafts in the setting of lost periorbital bone stock and minimizing foreign body in surgical fields at high risk of infection or complication. Further studies are necessary to refine the indications for autologous bone grafting and its benefit relative to alloplastic implants in ballistic periorbital trauma.

## Introduction

Orbital fractures are a common encounter for plastic and craniofacial surgeons, comprising 16% of all facial fractures [1]. Among these, orbital medial wall fractures can be technically difficult to repair and cause ophthalmic complications. The management of these fractures remains under debate due to the lack of clear consensus [2]. When repairing medial wall fractures, there are several aspects to consider, including the size of the defect, surgical approach, autologous versus alloplastic material, and which biomaterial to use for repair. Alloplastic biomaterials have largely replaced autologous grafts for orbital reconstruction, but despite the large variety of biomaterials available, there is still no agreement on the ideal implant for reconstruction [3-5]. Despite an evolution toward alloplastic implants, there may still be specific clinical indications for orbital reconstruction with autologous bone grafts.

In this case report, we present a 20-year-old male with a gunshot wound to the upper midface causing bilateral medial wall comminuted fractures, who subsequently underwent repair with split-thickness calvarial bone grafts through a preseptal transcaruncular transconjunctival approach. We discuss the consideration made when choosing how to repair this patient’s unusual type of fracture and why we used calvarial bone in a bilateral medial wall fracture.

## Case presentation

A healthy 20-year-old male presented to a regional trauma referral center intubated and sedated after sustaining a gunshot wound to the upper midface. There was an obvious entrance wound at the right medial canthus. Advanced trauma life support protocol was initiated. He was found to be hemodynamically stable and was taken to CT.

**Figure 1:**
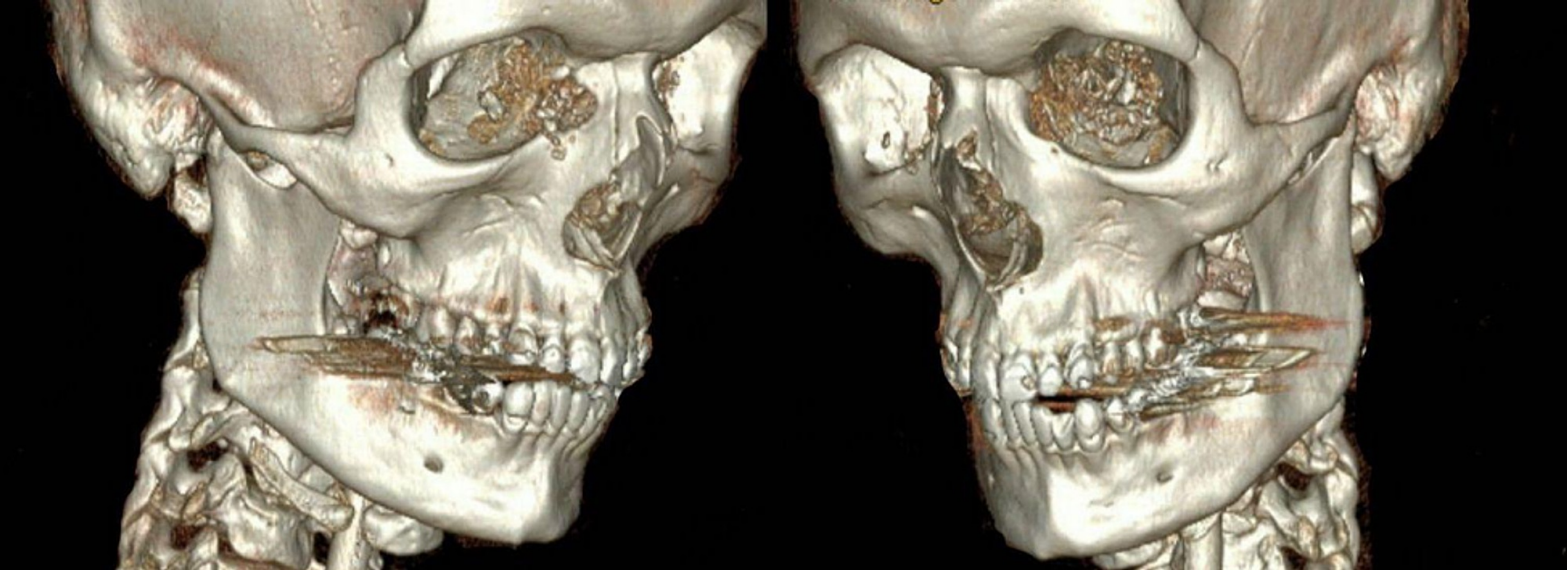
Three-dimensional reconstruction of patient’s admission computed tomography scan demonstrating severe comminution of medial orbital walls bilaterally

CT scan of the head and neck showed the path of the projectile going from right to left in a direction aimed inferiorly with the exit wound/fragments in the posterolateral left neck at the level of C3. It identified complete destruction of the laminae papyraceae, nasoethmoidal complexes, bony nasal septum, nasal turbinates, cribriform plates, and medial walls of the maxillary sinuses and the lateral wall of the left maxillary sinus. Large bony fragments and relatively large ballistic fragments were seen in the retrobulbar left orbit, but there was no entrapment of extraocular muscles (Figure 1).

He was admitted to the neurotrauma intensive care unit and consults were placed for ophthalmology and plastic surgery. On physical examination, there was a gunshot wound to the right medial canthal periorbital region, which was associated with left eyelid ptosis and bilateral periorbital ecchymosis and swelling. He had no evidence of entrapment, enophthalmos, or direct globe trauma. There was some concern for medial canthal tendon injury, but no evidence of hypertelorism. Given the possibility of optic nerve injury, it was important to avoid operative intervention in an acute setting while swelling was still present. Prior to surgical repair, an ophthalmic examination was completed. The patient was noted to have at least light perception vision after sedation was stopped in the early hospital course and displayed grossly intact visual acuity by discernment of the number of the examiner’s digits in all visual fields prior to orbital wall surgery. The patient’s right pupil was 3 mm, round, and minimally reactive. The left pupil was 4 mm, round, and subtly reactive. No relative afferent pupillary defect was noted. The dilated fundoscopic examination on the right showed a normal-appearing disc, macula, vessels, and periphery, while the left had mild foveal edema with a central dot hemorrhage. There was some subtle patchy Berlin’s edema surrounding the fovea with otherwise unremarkable optic discs, vessels, and peripheries bilaterally. Follow-up was recommended in the outpatient setting for further evaluation.

Surgical repair

**Figure 2:**
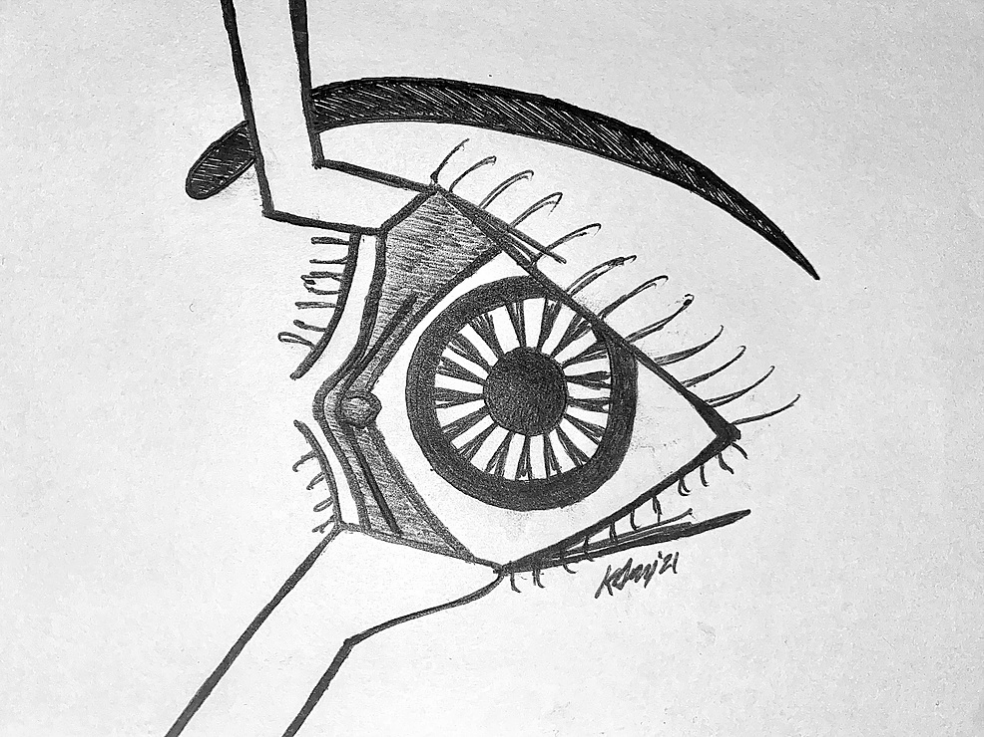
Trancaruncular transconjunctival incision

A transconjunctival approach with transcaruncular extension was performed bilaterally [6,7]. The lower eyelid was retracted and electrocautery was used to incise proximal to the tarsal vascular arcade of the lower palpebral conjunctiva medially between the lacrimal caruncle and plica semilunaris deep to the lacrimal canaliculi (Figure 2). This was followed by dissection superficial to the orbital septum to the arcus marginalis and then onto the orbital floor in a subperiosteal plane. The right medial canthal tendon was identified and appeared intact along with naso-orbital valley concavity, obviating transnasal wire reconstruction. Entry into the orbital floor was then performed with a periosteal elevator and progressed medially with the delivery of periorbital contents with the combination of an elevator and malleable retractor. The defect on the right measured approximately 2.8 x 2.0 cm while the left defect measured 1.8 x 2.0 cm. Once the defects were measured, attention was then turned to the cranial graft sites.

A superior coronal incision was made in a triangular pattern perpendicular to the hair follicles. Defect-matching bone grafts were designed and harvested with a fissure burr to the level of the diploe before a pineapple burr was used to taper the anterior non-harvested outer table for an angled 45° entry into the diploe with a curved 4 mm osteotome. A straight osteotome was used to harvest the remainder of the split-thickness calvarial grafts, which were then custom contoured using 1.2 mm eight-hole box plate fixation and in-fracture. The right-sided graft was placed with good bone-to-bone apposition at the anterior rim, taking care not to place the graft deeper than 30 mm into the medial orbital region to avoid optic nerve involvement. The graft was secured with a four-hole, low-profile miniplate with a single 3 mm screw to the medial orbital rim anteriorly and a single 3 mm screw to the graft posteriorly. A similar placement was achieved on the left side. The left-side graft was press-fit into place given less bony comminution and was stabilized by the surrounding periorbita, so screw fixation was not utilized. A similar placement was achieved on the left by a press fit with good stabilization from surrounding periorbita.

**Figure 3:**
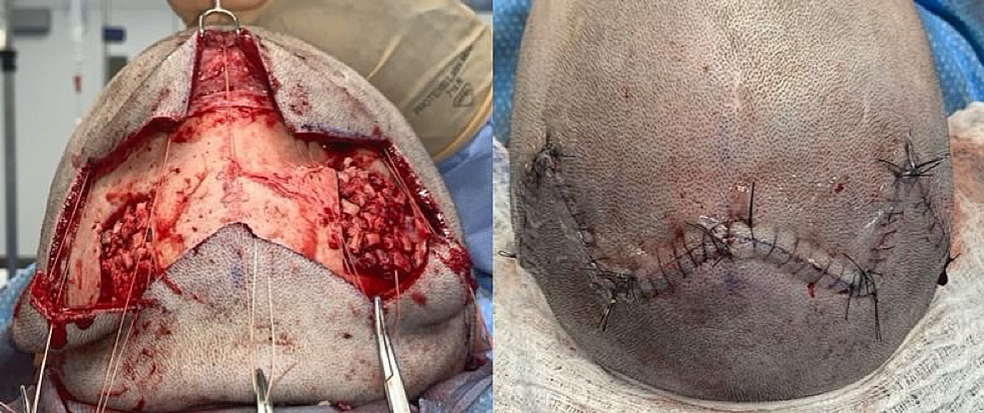
Intraoperative photos showing calvarial donor site (left), filled with morcellated excess bone chips and no depression in the scalp after closure (right)

All soft tissue layers were redraped over the grafts and forced duction testing performed bilaterally, showing unrestricted ocular excursion. A single stitch was placed in the inferior transconjunctival incision and medial to the transcaruncular region bilaterally. Temporary tarsorrhaphy stitches were placed at the inferior lid margins and secured to the forehead under gentle tension. Collected bone dust and morcellated bone chips were replaced in the graft sites to restore outer table contour (Figure 3, left) prior to redraping of the periosteum and reapproximation of galea. The scalp was then closed appropriately with deep dermal sutures as needed, interrupted vertical mattress sutures at each corner, and running sutures in each limb of the triangles (Figure 3, right).

Post-operative course

**Figure 4:**
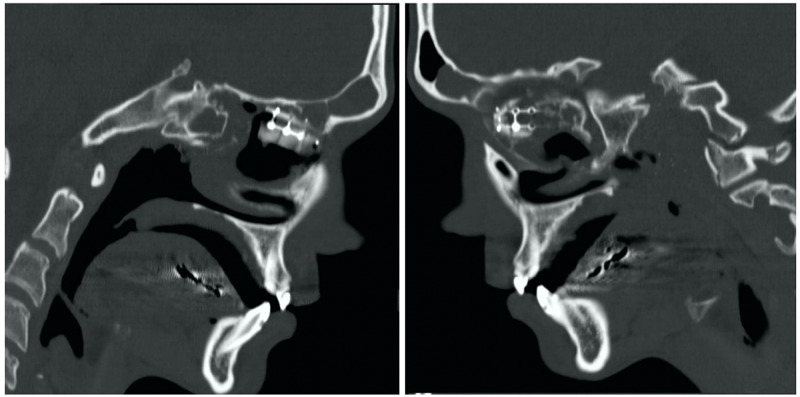
Sagittal computerized tomography showing the placement of graft on right (left) and left (right)

On postoperative day 1, the tarsorrhaphy stitches were removed at the bedside. CT scans were obtained on postoperative day 2 (Figure 4) and shown to have good orbital symmetry with defects repaired bilaterally, restoration of bone stock, and without periorbital tissue herniation or impingement. He continued preoperative antibiotics following surgery due to concomitant lobar pneumonia. 

The patient did well postoperatively and was discharged approximately one week later (following resolution of other non-maxillofacial conditions). On outpatient follow-up, he had no enophthalmos, exophthalmos, vertical dystopia, diplopia, blurred vision, or other visual issues. On physical examination, he had intact extraocular movements bilaterally, pupils were reactive to light and accommodation with grossly intact visual acuity. No proptosis or enophthalmos was noted, and he had a symmetric lid position with an equal scleral show. The donor site was well-healed with an incision concealed by hair regrowth and no contour defect or other problems. He continues to do well without complications one year postoperatively.

## Discussion

We describe a case of complex periorbital ballistic trauma with comminuted bilateral medial wall fractures with loss of bone stock. This projectile, shock-wave mechanism caused severe bony comminution with soft tissue maceration and a tract through the patient’s ethmoid air cells and nasopharyngeal tract to the contralateral skull base, raising concern for possible fistula formation and infection. Biocompatibility was therefore a priority, warranting autologous bone grafting with split-thickness calvarium to decrease graft resorption, donor site complications, and replenish deficient recipient site bone stock. Medial wall fractures are most commonly repaired with alloplastic implants, but implant-associated infection and extrusion risk were mitigated with autogenous grafting in this setting of communication between sensitive anatomic cavities. Graft contouring was accomplished by in-fracture and minimal titanium plating. Special clinical scenarios, such as ballistic trauma, may warrant the use of autologous reconstruction when bone stock is deficient and unnecessary foreign body material is contra-indicated.

Controversy remains about the ideal biomaterials for orbital reconstruction. Selection can be influenced by defect size, implant biocompatibility, donor site considerations, and ease of use. Autologous bone grafts used to be considered the gold standard for orbital repair due to their biocompatibility, stability, and strength [3,5,8]. Calvarial bone is typically the preferred donor site, due to donor site concealment, ease of accessibility, and lower resorption rates [8]. Rigid fixation allows faster tissue ingrowth, revascularization, and osteoconduction [3,8]. Drawbacks to autologous bone are donor site morbidity, variable resorption rates, and greater technical difficulty compared to alloplastic implantation [3,5-7,9-11]. Titanium is considered biocompatible, resistant to infection, and can osteo-integrate [3,5,8,9,11]. The titanium also offers strength and availability without donor site morbidity, making it ideal for large defects [3,8]. It is associated with a higher risk of infection and extrusion [5,9,11]. Despite these limitations, its use in orbital reconstruction has increased, while autologous bone graft use has diminished [3,9-11].

This case of complex medial orbital wall fractures due to ballistic trauma illustrates the preserved utility of autologous bone grafting, which successfully restored deficient midfacial bone stock while minimizing the risk of subsequent infection. We believe the associated donor site morbidity is therefore acceptable compared to the complication risk that an alloplastic reconstruction could have posed, though our report is limited by the retrospective nature of a single case report with intermediate follow-up. Autologous material should also possess a greater capacity for reorganization, another reason for use in the setting of severe trauma. Further studies will be required for more definitive conclusions to be drawn regarding the relative merit of autologous versus alloplastic reconstruction in ballistic maxillofacial trauma, but other literature supports the concepts employed in this case. Gosau et al. recommend that autologous bone be particularly considered for the repair of complex orbital fractures [10]. Pawar et al. suggest that ballistic orbital trauma reconstruction require liberal - if not exclusive - use of autologous bone grafts due to its superior incorporation into host bone, its lack of immune response, and less late extrusion [12]. The relative complication risks of alloplastic versus autologous implants may be negligible in more routine blunt trauma cases, but ballistic periorbital trauma may represent a scenario in which autologous reconstructive principles should be preserved. 

This case depicts the importance of tailoring biomaterial selection to individual clinical scenarios and illustrates the preserved utility of autologous orbital wall reconstruction following ballistic maxillofacial trauma.

## Conclusions

Ballistic maxillofacial injuries are highly destructive, posing unique reconstructive challenges. We report a case of an upper midface gunshot wound in a 20-year-old male producing bilateral, highly-comminuted medial orbital wall fractures. Reconstruction utilized bilateral split-thickness calvarial bone grafts through preseptal transcaruncular transconjunctival incisions. While orbital reconstruction has transitioned from primarily autologous grafts to alloplastic implants, this case illustrates the continued utility of autologous grafts to replenish bone stock and minimize foreign body in surgical fields at high risk of infection or implant extrusion. Further studies are necessary to refine the indications for autologous bone grafting and its benefit relative to alloplastic implants in ballistic periorbital trauma.
